# A Standardized Chemically Modified *Curcuma longa* Extract Modulates IRAK-MAPK Signaling in Inflammation and Potentiates Cytotoxicity

**DOI:** 10.3389/fphar.2016.00223

**Published:** 2016-07-25

**Authors:** Minakshi Rana, Preeti Maurya, Sukka S. Reddy, Vishal Singh, Hafsa Ahmad, Anil K. Dwivedi, Madhu Dikshit, Manoj K. Barthwal

**Affiliations:** ^1^Pharmacology Division, Council of Scientific and Industrial Research – Central Drug Research InstituteLucknow, India; ^2^Division of Pharmaceutics, Council of Scientific and Industrial Research – Central Drug Research InstituteLucknow, India

**Keywords:** caspase-3, chemically modified *Curcuma longa* extract, endotoxemia, interleukin-1 receptor-associated kinase 1, mitochondrial membrane potential

## Abstract

The TLR/IL-1R pathway is a critical signaling module that is misregulated in pathologies like inflammation and cancer. Extracts from turmeric (*Curcuma longa* L.) enriched in curcumin and carbonyls like turmerones have been shown to exert potent anti-inflammatory effects. The present study evaluated the anti-inflammatory activity, cytotoxic effect and the underlying mechanism of a novel chemically modified, non-carbonyl compound enriched *Curcuma longa* L. (*C. longa*) extract (CMCE). CMCE (1 or 10 μg/mL; 14 h) significantly decreased LPS (50-100 ng/mL) induced TNF-α and IL-1β production in THP-1 cells, human, and mouse whole blood as measured by ELISA. LPS-induced IRAK1, MAPK activation, TLR4 expression, TLR4-MyD88 interaction, and IκBα degradation were significantly reduced in CMCE pre-treated THP-1 cells as assessed by Western blotting. CMCE (30, 100, and 300 mg/kg; 10 days p.o.) pre-treated and LPS (10 mg/kg) challenged Swiss mice exhibited attenuated plasma TNF-α, IL-1β, nitrite, aortic iNOS expression, and vascular dysfunction. In a PI permeability assay, cell lines derived from acute myeloid leukemia were most sensitive to the cytotoxic effects of CMCE. Analysis of Sub-G1 phase, Annexin V-PI positivity, loss of mitochondrial membrane potential, increased caspase-3, and PARP-1 activation confirmed CMCE induced apoptosis in HL-60 cells. IRAK inhibition also sensitized HL-60 cells to CMCE induced cytotoxicity. The present study defines the mechanism underlying the action of CMCE and suggests a therapeutic potential for its use in sepsis and leukemia.

## Introduction

Chronic inflammation is associated with allergy, atherosclerosis, cancer, arthritis, and autoimmune disorders (Medzhitov, 2008). TLRs are well-known for their role in mediating signaling through pathogen-associated molecular patterns (PAMPs) of microbial products (Mogensen, 2009). Activation of TLR4 by LPS has been implicated in the pathophysiology of endotoxemic septic shock and lethality (Porter et al., 2010). LPS binding to TLR4 receptor induces MyD88 recruitment and subsequent activation of IRAK4. IRAK4 dependent IRAK1 and TNF receptor-associated factor 6 (TRAF6) activation induces NFκB transcription in an IκB kinase (IKK) dependent manner (Janssens and Beyaert, 2003). At the same time, IRAK1 can also induce the activation of the MAPK pathway (Janssens and Beyaert, 2003). NF-κB signaling and the MAPK-AP-1 axis often contribute to the secretion of cytokines and generation of an inflammatory response (Janssens and Beyaert, 2003; Palsson-McDermott and O’Neill, 2004).

Association of inflammation with development and progression of cancer depends on direct or indirect effects of cytokines, chemokines, growth factors, and receptors on tumor cells or their microenvironment (Balkwill and Mantovani, 2001; Kornblau et al., 2010). The aberrant regulation of cytokines and growth factors has also been observed in leukemias such as AML (Van Etten, 2007), suggesting the importance of inflammatory cytokine and chemokine antagonists in the prevention and treatment of malignant diseases (Balkwill and Mantovani, 2001). A recent study by Greten et al. (2004) demonstrated the role of NFκB in colitis-associated cancer using conditional inactivation of IKKβ in colonic epithelial cells. This resulted in a remarkable decrease in the tumor incidence (Greten et al., 2004). Overexpression and activation of IRAK1 and IRAK4 have been demonstrated in several types of cancer (Rhyasen and Starczynowski, 2015). Specifically, a role for IRAK in myeloid leukemia as well as in the progression of breast cancer has been shown recently (Rhyasen and Starczynowski, 2015; Wee et al., 2015). Epidemiological data suggests an inverse relationship between the occurrence of certain cancers and anti-inflammatory therapy (Rayburn et al., 2009).

Turmeric (*Curcuma longa L*.) has been traditionally used as an ayurvedic medicine and in the preparation of food (Prasad and Aggarwal, 2011). The bioactive components of *C. longa* are curcuminoids such as curcumin, demethoxycurcumin, bis-demethoxycurcumin and several volatile oils like d-α-phellandrene, d-sabinene, cinol, borneol, zingiberene, and sesquiterpenes (Prasad and Aggarwal, 2011). The oil derived from *C. longa* contains several carbonyl and non-carbonyl components like ar-turmerone, β-turmerone, α-turmerone, sesquiphellandrene, zingiberene, curcumene, curlone, germecrone, curzerene, curcumenol, iso-curcumenol, β-bisabolene, curdione, neocurdione, germacrene D, gamma-elemene, furanodieneone, furanodiene with trace amounts of curcumin, demethoxy curcumin, and bisdemethoxy curcumin (Ray et al., 2006). Previous reports have highlighted the anti-inflammatory effects of fractions enriched in curcumin and tumerones (Sandur et al., 2007; Bagad et al., 2013). However, the activity profile of the non-carbonyl components is not known.

Therefore, the present study was undertaken to investigate the potential anti-inflammatory and cytotoxic properties of a novel, chemically modified *C. longa* extract enriched in non-carbonyl components and to delineate the mechanism of action of this extract.

## Materials and Methods

### Materials

RevertAid^TM^ H Minus first strand cDNA synthesis kit was obtained from Fermentas Life Sciences (Vilnius, Lithuania). 2X Maxima SYBR Green RT-PCR Master Mix was purchased from Roche Applied Science (Lewes, UK). FBS and Penicillin/Streptomycin were obtained from Invitrogen (Carlsbad, CA, USA). Dexamethasone was purchased from EMD Millipore (Billerica, MA, USA). Antibodies against p-p38, p38, p-ERK1/2, ERK1/2, p-JNK1/2, JNK1/2, MyD88, Caspase-3, and IκBα were purchased from Cell Signaling Technology (Danvers, MA, USA). Anti-TLR4, anti-pIRAK1, anti-total IRAK1 and anti-cleaved PARP-1 were obtained from Santacruz Biotech Inc. (Santa Cruz, CA, USA). LPS (from *E. coli* 0111:B4), brewer TGA medium, 5,5′,6,6′-tetrachloro-1,1′,3,3′-tetraethylbenzimidazol carbocyanine iodide (JC-1), propidium iodide (PI), SRB, DOX and all other chemicals used in the present study were purchased from Sigma Chemicals, Co. (St. Louis, Mo, USA).

### Instrumentation

FTIR, NMR, and Mass were taken on a Perkin-Elmer Spectrum RX1 Spectrophotometer (4000-450 cm^-1^); (Waltham, MA, USA), Bruker Avance 400 (400 MHz FT-NMR with, 5 mm multi-nuclear inverse probe head, low and high-temperature facility and HRMAS accessory; USA) and Micromass Quattro II (USA), respectively.

### Preparation of CMCE Extract

A hexane soluble fraction of *C. longa* was prepared as described earlier (Rana et al., 2015). The hexane soluble fraction of *C. longa* (5 g) was mixed with semicarbazide hydrochloride (10 g) and ethyl alcohol (100 ml, 99% pure) with a few drops of glacial acetic acid, and then refluxed for 48 h. Further addition of semicarbazide hydrochloride (10 g) at an interval of 5 to 6 h was continued until the total amount of semicarbazide hydrochloride came up to 50 g. The completion of the reaction was monitored by TLC and finally by HPLC for the confirmation of complete removal of the carbonyl portion. The reaction mixture was cooled, filtered, the residual portion was washed with ethyl alcohol and the filtrate was concentrated under vacuum. Further HPLC grade hexane was added to the concentrate and refluxed for 5 h for the extraction of *Curcuma longa* L. extract (CMCE) from ethanol. It was filtered again and concentrated under vacuum. No crystal formation was observed on retaining the CMCE.

### Cell Culture and Treatments

THP-1 cells (Human monocytic leukemia), HL-60 cells (Human Caucasian promyelocytic leukemia), MCF-7, and MDA-MB-231 (Human Caucasian breast adenocarcinoma) were obtained from American Type Culture Collection (Rockville, MD, USA). THP-1 and HL-60 cells were cultured in RPMI-1640 medium while MCF-7 and MDA-MB-231 cells were grown in DMEM containing 10% heat-inactivated FBS, 100 IU/mL penicillin, and 100 μg/mL streptomycin. THP-1 cells were pre-treated with CMCE (1 or 10 μg/mL) or dexamethasone (standard anti-inflammatory drug; 1 μg/mL) for 14 h followed by LPS (100 ng/mL) stimulation for 1.5 h. Experiments related to human blood were carried out after receiving proper ethics approval from the Institute, King George’s Medical University (KGMC) human research ethics committee. Human blood was collected from the antecubital vein in heparin tubes (Vacutainer) at the department of transfusion medicine, KGMC after receiving prior permission from healthy volunteers. Peripheral blood mononuclear cells (PBMCs) were isolated using percoll density gradient centrifugation as described previously (Repnik et al., 2003; Tiwari et al., 2011). All ethical procedures were in accordance with the Declaration of Helsinki. For cytotoxicity assays, HL-60, MCF-7, and MDA-MB-231 cells and human PBMCs were treated with CMCE (1-100 μg/mL) or commercial anti-tumor standard drug DOX (1 μg/mL) for 20 h. CMCE or standard drugs were dissolved in DMSO (0.1% (v/v) vehicle) for cell culture experiments. The vehicle control had no effect on cytotoxicity (data not shown).

### Whole Blood Culture

Blood from anesthetized male Swiss albino mouse was collected by cardiac puncture in 2.5% trisodium citrate under aseptic conditions in accordance with the institutional guidelines under an approved ethical protocol. Human and mouse whole blood was diluted 1:10 in RPMI-1640 and treated with CMCE (1 and 10 μg/mL) or dexamethasone (1 μg/mL) for 14 h followed by LPS (50 ng/mL) treatment for 8 h. TNF-α and IL-1β in the culture supernatants were measured using ELISA (Thurm and Halsey, 2005).

### Cell Viability

#### PI Permeability

THP-1, HL-60, MCF-7, MDA-MB-231 cells, and human PBMCs (1 × 10^6^ cells/mL) treated with CMCE were incubated with PI (5 μg/mL in PBS) for 5 min at room temperature, and then a minimum of 10,000 events were acquired by flow cytometry and subsequently analyzed using the CellQuest program (FACS Calibur; Becton-Dickinson, San Jose, CA, USA; Kumar et al., 2010a).

#### SRB Assay

Cell viability was determined using the SRB method as described previously (Skehan et al., 1990). Briefly, HL-60 cells were fixed with 80% TCA (w/v) for 1 h at 4°C. The fixed cells were washed five times with tap water, air-dried and stained with SRB solution 0.4% (w/v) in 1% acetic acid for 30 min at room temperature. After incubation, excess SRB was removed by washing four times with 1% acetic acid and air dried. 10 mM Tris-base (pH 10.5) was added to each well to solubilize SRB by shaking the plate for 5 min on a shaker, and the absorbance of SRB was measured at 564 nm by ELISA plate reader (BioTek Instrument Inc., Winooski, VT, USA).

### Cytokine Measurement

The levels of cytokines (TNF-α and IL-1β) were measured using conventional ELISA kits (BD OptEIA^TM^ set; BD Biosciences, San Diego, CA, USA) according to the manufacturer’s instructions (Tiwari et al., 2011).

### Nitrite Measurement

Total nitrite content in the plasma and cell culture supernatant was measured by the Griess reagent. To estimate nitrite content in plasma and supernatant, cadmium pellets (Sigma, St. Louis, MO, USA) were added to reduce nitrate to nitrite. An equal volume of Griess reagent was added to the respective samples and incubated for 30 min at 37°C. Nitrite concentration was estimated by measuring the absorbance at 545 nm and 630 nm (wavelength correction) against sodium nitrite as standard using ELISA plate reader (Biotek Instrument Inc., Winooski, VT, USA). Total nitrite was reported as percent (%) nitrite release (Kumar et al., 2010b; Rana et al., 2015).

### Western Blotting and Co-immunoprecipitation

Whole cell extracts from THP-1 and HL-60 cells after various treatments were prepared in lysis buffer (0.01 M Tris-HCl (pH 7.4), 0.1 M NaCl, 0.001 M EDTA (pH 7.4), aprotinin (1 μg/mL), phenylmethylsulfonyl fluoride (100 μg/mL), pepstatin (20 μg/mL), sodium orthovanadate (Na_2_VO_4_, 2 mM), sodium fluoride (2 mM) and 1% Triton X-100). THP-1 or HL-60 cell lysates were centrifuged at 13,000 × *g* for 10 min and protein concentrations were measured by using BCA reagent. For co-immunoprecipitation, Sepharose A beads (20 μL) were conjugated with anti-TLR4 (1 μg) using a pre-adsorption buffer (50 mM HEPES; pH 7.4, 150 mM NaCl, and 1% Triton X-100) for 3 h at 4°C on rotating platform followed by a single wash with cold PBS. Protein (500 μg) was added to TLR4 conjugated beads and incubated overnight at 4°C. Subsequently, protein or immunoprecipitates were separated on 8-10% SDS-PAGE and transferred to PVDF membrane. After blocking with 5% BSA in TBST, the membranes were probed with primary antibody against various proteins of interest. The specific bands were detected by enhanced chemiluminescence (Tiwari et al., 2011). Western blots results were expressed as fold change in relative image quant units in comparison to control. Protein phosphorylation was normalized against their respective total protein expression and loading control. Densitometry quantification of the immunoprecipitation blots was normalized after considering the total input expression of the respective protein.

### LPS-Induced Endotoxemia Mouse Model

Male Swiss albino mice (20-25 g; 8 weeks old) obtained from the National Laboratory Animal Centre at the CSIR-Central Drug Research Institute were used in this study as per Institutional Animal Ethics Committee guidelines. In the present study, male mice were used since they were readily available at the animal house facility and this also helped in avoiding interference of the female hormones, if any, on the studied parameters. All animal procedures were followed in accordance with Institutional Animal Ethics Committee, which follows the guidelines of the Committee for the Purpose of Control and Supervision of Experiments on Animals and conforms to the Indian National Science Academy international norms. After acclimatization, mice were randomly grouped into six groups of six animals each; Control (vehicle; group I), LPS (10 mg/kg; group II), CMCE (30, 100, and 300 mg/kg; group III, IV and V, respectively) and dexamethasone (10 mg/kg; group VI). CMCE was administered via oral gavage in 0.25% carboxymethyl cellulose sodium suspension (CMC; vehicle) for 10 days prior to LPS (10 mg/kg; i.p.) challenge given for 12 h (Schmidt et al., 2007). Dexamethasone was injected intraperitoneally 1 h before LPS (10 mg/kg; i.p.) challenge (Schmidt et al., 2007). No mortality was observed after LPS administration. The dose selected for LPS administration is on the lines of reported dose where mortality was also not observed (Xu et al., 2012).

### TGA-Induced Peritonitis Mouse Model

Male Swiss albino mice (20-25 g; 8-10 weeks old) were randomly distributed into six groups of four animals each; vehicle control (0.25% CMC w/v; group I), TGA (4%; group II; i.p. 24 and 72 h), CMCE (30, 100, or 300 mg/kg; group III, IV, and V, respectively; p.o. 10 days) and dexamethasone (1.5 mg/kg; group VI; i.p. 1 h) (Montesinos et al., 2006). Peritoneal fluid containing leukocytes was collected at 24 and 72 h after TGA injection by flushing the peritoneal cavity with 5 mL of cold PBS. The number of cells recruited into the peritoneal cavity was determined with a Neubauer chamber (Rana et al., 2015).

### RNA Isolation and Real-Time RT-PCR

Total RNA was extracted from mouse aortic tissue by TRIzol reagent (Invitrogen, Carlsbad, CA, USA) and cDNA was prepared using the RevertAid^TM^ H Minus first strand cDNA synthesis kit (Fermentas, Vilnius, Lithuania). Quantification of mRNA by real-time PCR was carried out using the Light Cycler^®^ 480II Real-Time PCR system (Roche Applied Science, Lewes, East Sussex, UK) along with 2X Maxima SYBR Green RT-PCR Master Mix. The cDNA was amplified using the following primers: forward 5′-TGCATGGACCAGTATAAGGCAAGC-3′ and reverse 5′-CTCCTGCCCACTGAGTTCGTC-3′ for iNOS; and forward 5′-CGTTGACATCCGTAAAGACC-3′ and reverse 5′-TGGAGCCACCGATCCACACA-3′ for β-Actin. Samples were incubated at 95°C for 5 min, followed by 45 cycles, each consisting of a 15 s denaturation at 95°C, annealing at 59°C for 20 s, and extension at 72°C for 15 s. Relative fold difference between the groups was calculated by using the comparative cycle threshold (2^-ΔΔCt^) method. β-Actin was used as an internal standard to calculate the relative expression (Rana et al., 2015).

### Endothelial Function

Vasoreactivity was monitored in the thoracic aorta of LPS and CMCE treated mice as described earlier (Singh et al., 2011). Briefly, thoracic aorta was cut and mounted in organ baths containing 10 mL Krebs bicarbonate solution. Changes in isometric force were amplified via Compact Research System Amplifiers and were recorded with force transducers (FSG-01; Budapest, Hungary) using SPEL Solution Pack for Experimental Laboratories ADVANCE ISOSYS data acquisition and analysis software. After equilibration, the aortic rings were exposed to KCl Krebs buffer (80 mM) to assess the maximum tissue contractility. Cumulative concentration-dependent contraction responses to phenylephrine (PE) (10-100 μM) were assessed. The endothelium function was then assessed by monitoring relaxation to acetylcholine (Ach) (300 pM-300 μM) in PE (1 μM) pre-contracted rings. Finally, tissue contractility and viability were assessed by exposing the rings to KCl Krebs buffer (80 mM) in all groups (Singh et al., 2011).

### Bioavailability Studies

Preparation of standard and quality control samples was done (Supplementary Material). CMCE was administered orally at a dose of 300 mg/kg in mice. Blood samples were collected at pre-defined time intervals (5, 15, 30, 60, 120, 180, 240, 360, and 1440 min; *n* = 3). Plasma was harvested by centrifuging the blood at 4500 × *g* for 10 min. To 0.2 mL of plasma, 0.5 mL of methanol was added and thoroughly vortex mixed for 2 min. After centrifugation at 3000 × *g* for 10 min, the supernatant layer was transferred into a clean test tube, concentrated to dryness under vacuum, reconstituted in 50 μL of methanol, 20 μL of which were injected into the HPLC system (Shimadzu, Kyoto, Japan) for analysis (Jain et al., 2007).

### Cell Cycle and Apoptosis

The DNA staining in HL-60 cells (1 × 10^6^ cells/mL) was performed using hypotonic PI solution (50 μg/mL PI with 0.03% NP-40 in 0.1% sodium citrate) as described earlier (Krishan, 1975). The cells undergoing apoptosis were obtained from the sub-G1 region of the DNA distribution histograms. Analysis of cellular apoptosis was assessed using the Annexin V-FITC/PI kit (BD Biosciences, San Jose, CA, USA). Stained cells were acquired by flow cytometer and analyzed using CellQuest software (Becton Dickinson, San Jose, CA, USA) in the case of apoptotic markers or by Modfit software (Verity Software, Topsham, ME, USA) for the cell cycle analysis. PARP-1 and caspase-3 activation were monitored as markers of apoptosis in HL-60 cells by Western blotting.

### Mitochondrial Membrane Potential

HL-60 cells were stained with JC-1 (5 μM) in RPMI-1640 for 10 min as previously described (Kumar et al., 2010a). Cells were subsequently washed twice with PBS, and the pellet was re-suspended in PBS for FACS analysis. A minimum of 10,000 events was acquired to assess JC-1 fluorescence in both FL-1 (monomers, green fluorescence) and FL-2 channels (aggregates, red fluorescence) using FACS Calibur (Becton-Dickinson, San Jose, CA, USA).

## Statistical Analysis

Results are expressed as the mean ± standard error (S.E). The statistical differences between groups were determined by one-way ANOVA followed by Tukey’s or Dunnett’s *post hoc* test, and *p*-value equal to or less than 0.05 was considered as statistically significant. All statistical analysis were performed with the GraphPad Prism 5.0 program (GraphPad Inc., La Jolla, CA, USA).

## Results

### CMCE Reduces Pro-Inflammatory Cytokine Production in LPS-Treated THP-1 Cells and Whole Blood from Human and Mouse

A cell viability test was performed at 14 h in THP-1 cells to exclude toxic doses of CMCE. CMCE reduced cell viability by ∼73% at 30 μg/mL and ∼93% at 100 μg/mL doses as measured by the PI permeability assay while no significant cytotoxicity was observed at lower doses (1 and 10 μg/mL) as compared control cells (Supplementary Figure S1). Therefore, these lower doses of CMCE were further assessed in THP-1 cells as well as in human and mouse whole blood to determine the effects on LPS-induced TNF-α and IL-1β production. Pre-treatment with CMCE (1 and 10 μg/mL) for 14 h decreased LPS-induced TNF-α (∼70 and 76%, respectively) and IL-1β (∼49 and 96%, respectively) production in THP-1 cells (**Table 1**). This effect of CMCE was comparable to the effect of the known anti-inflammatory agent, dexamethasone, which reduced LPS-induced TNF-α and IL-1β (∼77 and 50%, respectively) production in THP-1 cells (**Table 1**). To further these results, we tested the effect of CMCE in whole blood derived from mouse or human, a physiologically more relevant system. CMCE pre-treatment (1 and 10 μg/mL) dose-dependently decreased LPS-induced TNF-α production in human (∼17 and 46%, respectively) and mouse (∼16 and 45%, respectively) whole blood (**Table 1**). CMCE (1 and 10 μg/mL) also decreased IL-1β production in human (∼17 and 57%, respectively) and mouse (∼24 and 64%, respectively) whole blood (**Table 1**). Dexamethasone showed a similar reduction in TNF-α and IL-1β production in blood cells (∼59 and 67%, respectively) (**Table 1**).

**Table 1:** *Curcuma longa* L. extraxt (CMCE) reduces LPS-induced pro-inflammatory cytokines production in THP-1 cells, human and mouse whole blood.

	Groups	TNF-α (ρg/mL)	IL-1β (ρg/mL)
	Control	11.6 ± 2.7	6.8 ± 2.1
	LPS	3374 ± 357.5^∗∗∗^	198.6 ± 39.9^∗∗∗^
THP-1 cells	LPS+CMCE 1 μg/mL	1016 ± 150.6^###^	101.5 ± 2.1^#^
	LPS+CMCE 10 μg/mL	826.1 ± 213.7^###^	8.7 ± 0.2^###^
	LPS+Dexamethasone 1 μg/mL	762.9 ± 136.4^###^	99.4 ± 4.3^#^
	Control	27.4 ± 3.7	14.1 ± 5.1
	LPS	1691 ± 120.2^∗∗∗^	1040 ± 60^∗∗∗^
Human whole blood	LPS+CMCE 1 μg/mL	1397 ± 115.6	864.4 ± 43.4
	LPS+CMCE 10 μg/mL	911 ± 54.6^###^	439.6 ± 56.5^###^
	LPS+Dexamethasone 1 μg/mL	699.2 ± 67.9^###^	343.4 ± 45^###^
	Control	109.1 ± 21.1	121.8 ± 16.4
	LPS	431.8 ± 22^∗∗∗^	416.5 ± 18.7^∗∗∗^
Mouse whole blood	LPS+CMCE 1 μg/mL	361.3 ± 26	313.5 ± 44.1
	LPS+CMCE 10 μg/mL	236 ± 11.3^###^	150 ± 31.5^###^
	LPS+Dexamethasone 1 μg/mL	174.7 ± 21.5^###^	138.8 ± 32.5^###^

*Data were expressed as the mean (at least *n* = 3) ± SEM; ^∗∗∗^*p* < 0.001 control vs. LPS; ^#^*p* < 0.05, ^###^*p* < 0.001 LPS vs. LPS+CMCE or LPS+dexamethasone.*

### CMCE Inhibits Activation of IRAK1 and MAPKs in LPS-Stimulated THP-1 Cells

**FIGURE 1:**
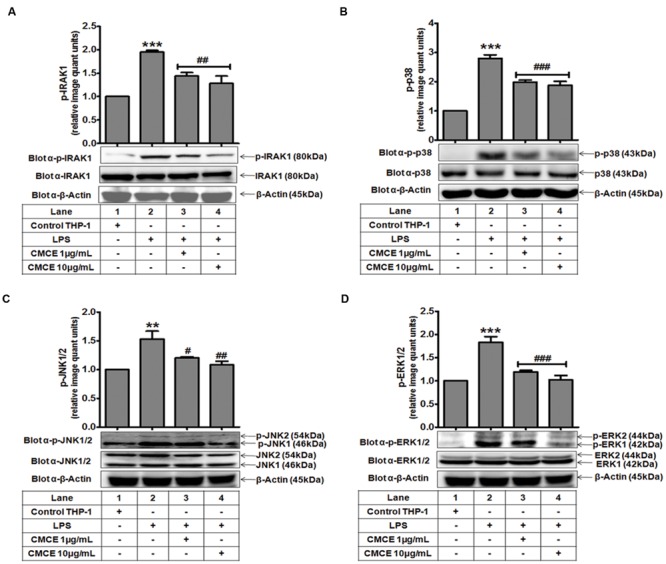
**The effect of *Curcuma longa* L. extract (CMCE) on LPS-induced IRAK1 and MAPKs activation.** Phosphorylation and expression of **(A)** IRAK1, **(B)** p38, **(C)** JNK, and **(D)** ERK MAPK in CMCE pre-treated (1 and 10 μg/mL for 14 h) and LPS stimulated (100 ng/mL for 1.5 h) THP-1 cells. β-Actin was used as an internal loading control. Values represent the mean ± SEM; ^∗∗^*p* < 0.01, ^∗∗∗^*p* < 0.001 control vs. LPS; ^#^*p* < 0.05, ^##^*p* < 0.01, ^###^*p* < 0.001 LPS vs. LPS and CMCE pre-treated cells. The blots are representative one of three similar experiments.

Mitogen-activated protein kinases (p38, JNK, and ERK) often operate downstream of the TLR-IRAK pathway and are intimately involved in cytokine production (Huang et al., 2004). Therefore, we examined phosphorylation of IRAK1 and MAPKs in LPS stimulated THP-1 cells. LPS-induced IRAK1 phosphorylation in THP-1 cells (∼1.9 fold) was significantly attenuated in CMCE (1 and 10 μg/mL; 14 h) pre-treated cells (∼1.4 and 1.5 fold, respectively) (**Figure 1A**). Further, LPS significantly augmented phosphorylation of p38 (∼2.8 fold), JNK1/2 (∼1.5 fold) and ERK1/2 (∼1.8 fold) in THP-1 cells (**Figures 1B–D**). However, CMCE (1 and 10 μg/mL) pre-treated THP-1 cells showed marked reduction in LPS-induced p38 (∼1.4 and 1.5 fold, respectively; **Figure 1B**), JNK1/2 (**∼**1.3 and 1.4 fold, respectively, **Figure 1C**) and ERK1/2 (**∼**1.5 and 1.8 fold, respectively; **Figure 1D**) phosphorylation as compared to cells stimulated with LPS alone.

### CMCE Attenuates the LPS-Induced TLR4-MyD88 Interaction, TLR4 Expression and IκBα Degradation in THP-1 Cells

**FIGURE 2:**
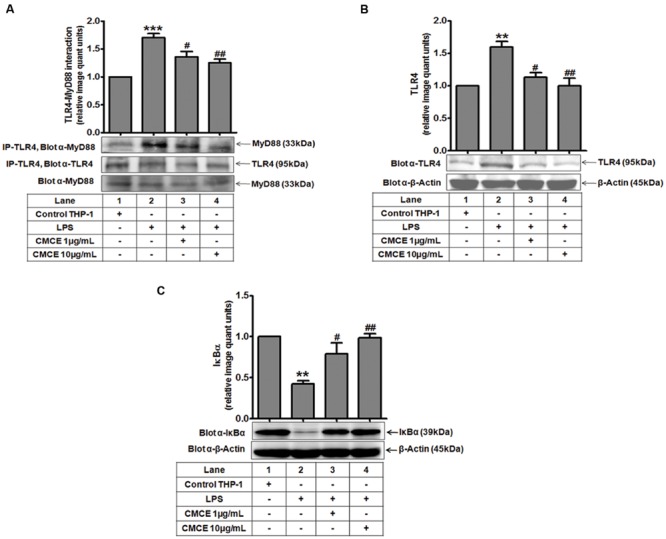
**The effects of CMCE on LPS-induced TLR4-MyD88 interaction, TLR4 expression, and IκBα degradation. (A)** TLR4-MyD88 interaction in THP-1 cells pre-treated with CMCE (1 and 10 μg/mL; 14 h) and stimulated with LPS (100 ng/mL; 5 min). Cell lysates were immunoprecipitated with anti-TLR4 and immunoblotted with MyD88. Expression of **(B)** TLR4 and **(C)** IκBα in THP-1 cells pre-treated with CMCE (1 and 10 μg/mL; 14 h) and further challenged with LPS (100 ng/mL; 1.5 h). β-Actin was used as an internal loading control. Values represent the mean ± SEM; ^∗∗^*p* < 0.01, ^∗∗∗^*p* < 0.001 control vs. LPS; ^#^*p* < 0.05, ^##^*p* < 0.01 LPS vs. LPS and CMCE pre-treated cells. The blots are representative one of three similar experiments.

TLR4 triggers the downstream signaling pathway by recruiting the MyD88 adaptor protein to its receptor (Medvedev et al., 2002). Expression of TLR4 after LPS stimulation was monitored in a time-dependent manner. As shown earlier (Muzio et al., 1998; Medvedev et al., 2002), LPS-induced TLR4 expression was unchanged as compared to control cells at early time points (5 min, 15 min); however it was significantly augmented at later time points (1, 1.5, and 2 h) (data not shown). In contrast, TLR4-MyD88 complex formation was significantly induced (∼1.7 fold) after 5 min of LPS stimulation of THP-1 cells (**Figure 2A**), which was significantly attenuated following CMCE (1 and 10 μg/mL) pre-treatment (∼1.3 and 1.4 fold, respectively) (**Figure 2A**). Interestingly, expression of TLR4 and MyD88 was unaltered at this time point (**Figure 2A**). Similarly, LPS-induced TLR4 expression, which was significantly augmented (∼1.6 fold) at later time point (1.5 h) (**Figure 2B**), was also decreased in CMCE (1 and 10 μg/mL) pre-treated THP-1 (∼1.4 and 1.6 fold, respectively) cells (**Figure 2B**). We next examined the effect of CMCE on LPS-induced IκBα degradation in THP-1 cells. LPS-induced IκBα degradation (∼0.4 fold) was recovered in CMCE (1 and 10 μg/mL) pre-treated THP-1 cells in a dose-dependent manner (∼1.8 and 2.3 fold, respectively) (**Figure 2C**).

### CMCE Prevents LPS-Induced Adverse Effects in Mice

**FIGURE 3:**
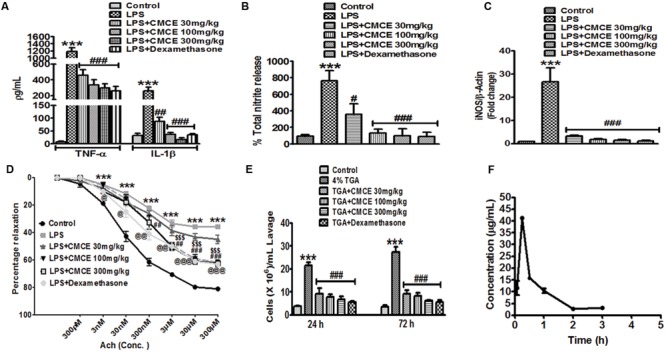
**The effects of CMCE in mice.** Plasma **(A)** TNF-α, IL-1β and **(B)** nitrite production, **(C)** aortic iNOS mRNA expression, and **(D)** endothelial dysfunction in CMCE (30, 100, and 300 mg/kg; 10 days p.o.) pre-treated and LPS- challenged (10 mg/kg; 12 h) male Swiss mice. **(E)** Inflammatory cell migration in CMCE (30, 100, and 300 mg/kg; 10 days p.o.) or dexamethasone (1.5 mg/kg; 1 h i.p.) pre-treated and 4% TGA- challenged mice. Migration of leukocytes was counted after 24 and 72 h of TGA challenge using Neubauer chamber. **(F)** Plasma concentration time profile of Compound (I). Values represent the mean (*n* = 3-6) ± SEM; ^∗∗∗^*p* < 0.001 control vs. LPS or TGA; ^#^*p* < 0.05, ^##^*p* < 0.01, ^###^*p* < 0.001, ^$$$^*p* < 0.001, ^@^*p* < 0.05, ^@@^*p* < 0.01, ^@@@^*p* < 0.001 LPS vs. LPS+CMCE or dexamethasone pre-treated groups.

Plasma levels of TNF-α, IL-1β, and total nitrite were significantly (*p* < 0.001) increased in mice 12 h following LPS challenge (10 mg/kg; **Figures 3A,B**). However, mice that were orally gavaged with CMCE (30, 100, 300 mg/kg) daily for 10 days prior to LPS challenge showed a significant reduction in LPS-induced TNF-α (∼61, 71 and 75%, respectively), IL-1β (∼66, 86, and 93%, respectively) and nitrite production (∼53, 83, and 87%, respectively) (**Figures 3A,B**). These levels were comparable to that seen with a 10 mg/kg dosage of dexamethasone administered intraperitoneally to animals 1 h prior to LPS challenge. Dexamethasone (10 mg/kg) pre-treated mice also showed an inhibition of plasma TNF-α, IL-1β and nitrite production (∼78, 87, and 88%, respectively) (**Figures 3A,B**). Furthermore, increased aortic iNOS expression (∼27 fold) in LPS-challenged mice (**Figure 3C**) was significantly attenuated in CMCE (30, 100, and 300 mg/kg) pre-treated mice (∼3, 1.9, and 1.4 fold, respectively) as compared to the control group (**Figure 3C**). Mice pre-treated with dexamethasone (10 mg/kg) also showed attenuated iNOS expression (∼1.1 fold) (**Figure 3C**). Because of the role of iNOS in endothelial dysfunction is well-characterized (Titheradge, 1999; Draisma et al., 2010), we sought to determine whether CMCE may mitigate this response via its attenuation of iNOS expression. A significant (*p* < 0.001) reduction in acetylcholine-induced endothelial relaxation in PE pre-contracted rings was observed in the aorta from the LPS treated group when compared to the control group (**Figure 3D**). However, no change was seen in PE-induced contractions. CMCE (100 and 300 mg/kg) or dexamethasone pre-treatment (10 mg/kg) of LPS-challenged mice, significantly (*p* < 0.01, *p* < 0.001) restored acetylcholine-induced endothelial relaxation (**Figure 3D**). However, no change was observed at a lower dose of CMCE (30 mg/kg).

To determine the effect of CMCE on inflammatory cell migration *in vivo*, we studied the recruitment of cells into the peritoneal cavity elicited by TGA. In particular, we observed the expected increase in leukocytes found in peritoneal lavage (21 × 10^6^ cells/ mL lavage fluid; *p* < 0.001 at 24 h and 27 × 10^6^ cells/ mL lavage fluid; *p* < 0.001 at 72 h, respectively) after TGA injection (**Figure 3E**). As predicted, CMCE (30,100 and 300 mg/kg) pre-treatment significantly attenuated transmigration of these cells (9 × 10^6^, 8 × 10^6^, and 7 × 10^6^ cells/ mL lavage fluid; *p* < 0.001, respectively) at 24 h and (9 × 10^6^, 8 × 10^6^, and 6 × 10^6^ cells/ mL lavage fluid; *p* < 0.001, respectively) at 72 h in the peritoneal cavity of TGA-challenged mice. Similarly, dexamethasone-treated mice showed reduced leukocyte influx into peritoneal cavity at 24 and 72 h (6 × 10^6^ cells/mL lavage fluid; *p* < 0.001, respectively) as compared to the TGA group (**Figure 3E**).

### Bioavailability Studies

In our preparation, the yield of CMCE was 3 g (40%). The constituents of CMCE included zingiberine, curcumene, β-bisabolene, β-sesquiphellandrene, curzerene, and a cyclic derivative of the carbonyl compound 7,7-dimethyl-5-(2-p-tolylpropyl)-6,7-dihydro-1,3,4-oxadiazepin-2-amine, compound (I). The latter was identified and characterized as the major marker compound present in CMCE, using ^1^H, ^13^C NMR, and IR spectral analysis and mass spectrometry (Supplementary Figure S2). Pharmacokinetic parameters were determined based on the presence of compound (I) in CMCE, which was not less than 25%. Its purity was ascertained by TLC and HPLC, and it was found to be more than 99% pure. Therefore, the concentration of marker compound (I) was utilized to assess the bioavailability of CMCE using plasma extracted from mice orally gavaged with CMCE. The HPLC chromatogram of blank plasma and plasma spiked with CMCE in which the marker compound (I) was eluted at about 4.6 min (Supplementary Figure S3). The HPLC chromatogram of plasma after 15 min of CMCE administration showed compound (I) (Supplementary Figure S4). All subjects showed early absorption of marker compound (I), which appeared to peak at 15 min with plasma levels of (I) measuring 41.29 ± 1.07 μg/mL (**Figure 3F**). By 240 min, the plasma concentration of compound (I) was below the detection limit, indicative of rapid absorption and elimination of the marker compound. Overall systemic availability of (I) was found to be 32.65 ± 3.32 h^∗^μg/mL (equivalent to CMCE).

### Comparison of Efficacy of CMCE in HL-60, MCF-7, MDA-MB-231 Cells, and PBMCs

**FIGURE 4:**
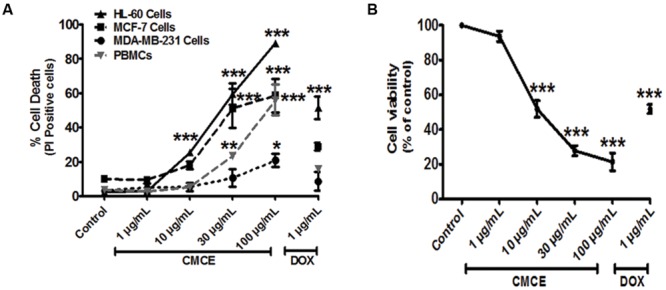
**Effects of CMCE on cell viability of HL-60, MCF-7, MDA-MB-231 cells, and PBMCs.** The cells were treated with different concentrations of CMCE (1, 10, 30, and 100 μg/mL) or DOX (1 μg/mL) for 20 h and cell viability was determined by **(A)** PI permeability in HL-60, MCF-7, MDA-MB-231 cells, and PBMCs **(B)** SRB assay in HL-60 cells. Values represent the mean (at least *n* = 3) ± SEM; ^∗^*p* < 0.05, ^∗∗^*p* < 0.01, ^∗∗∗^*p* < 0.001 control vs. CMCE or DOX treated cells.

Since altered IRAK signaling has been associated with the development of myeloid leukemia and breast cancer and since we demonstrate here that CMCE modulates IRAK signaling in THP-1 AML cells, we hypothesized that CMCE might show cytotoxic effects in AML- and breast cancer-derived cell lines. To that end, HL-60, MCF-7, and MDA-MB-231 cells were treated with 1, 10, 30, or 100 μg/mL CMCE. A concentration-dependent effect on cytotoxicity was noted. However, the most robust anti-proliferative effect of CMCE leading to a maximum number of PI-positive cells was observed in HL-60 cells (**Figure 4A**). The order of CMCE sensitivity as demonstrated in term of PI positivity was MDA-MB-231 cells < MCF-7 cells < HL-60 cells. Mechanistic studies were carried out in HL-60 cells since CMCE induced significantly more apoptosis in this cell line when compared to other solid tumor cells like MCF-7 and MDA-MB-231. To determine whether CMCE selectively acted on cancer cells, primary human PBMCs were used as healthy, non-transformed controls. CMCE (10 μg/mL) induced cell death (∼25%) in HL-60 cells after 20 h, while no significant cytotoxicity was observed at this dose in normal PBMCs (**Figure 4A**). Since the extent of cell death induced by CMCE at a lower dose (10 μg/mL) was significantly less in healthy PBMCs when compared to HL-60 cells, it can be speculated that CMCE may produce additional beneficial effects by targeting the cancer cells more effectively than the healthy ones. However, at higher doses a significant increase in cell death was observed in PBMCs (**Figure 4A**). As expected, DOX, a therapeutic commonly used in the treatment of hematological malignancies, reduced the cell viability to an extent similar to that seen with the 30 μg/mL dosage of CMCE (∼51% vs. 59%) in HL-60 cells using the PI assay. This cytotoxicity was independently confirmed using an SRB assay, which is currently employed in the NCI-60 cell line screen assay (Skehan et al., 1990). A dose-dependent (1, 10, 30, and 100 μg/ml) inhibitory effect on cell proliferation was observed (∼6, 48, 72, and 78%, respectively) in CMCE-treated HL-60 cells. This was comparable to the 48% inhibition of proliferation seen in HL-60 cells treated with 1 μg/mL of DOX (**Figure 4B**).

### CMCE Induces Formation of an Apoptotic Sub-G1 Peak and Phosphatidylserine Externalization in HL-60 Cells

**FIGURE 5:**
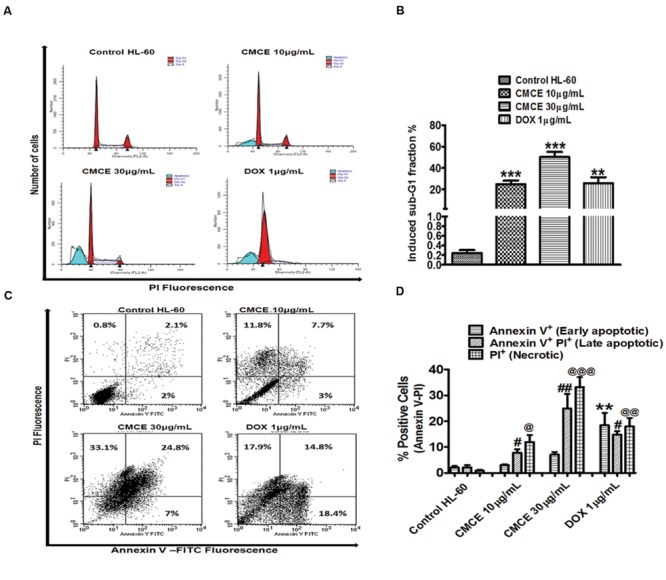
**Effect of CMCE on Sub-G1 apoptosis and phosphatidylserine externalization. (A)** Histogram and **(B)** bar diagram represent sub-G1 fraction as analyzed by flow cytometer using hypotonic PI and **(C)** flow cytometric dot plot, **(D)** bar diagram representing Annexin V-PI staining in HL-60 cells treated with CMCE (10 and 30 μg/mL) or DOX (1 μg/mL) for 20 h. Values represent the mean ± SEM; ^∗∗^*p* < 0.01, ^∗∗∗^*p* < 0.001 control vs. CMCE or DOX; ^∗∗^*p* < 0.01 control Annexin V^+^ PI^-^ cells vs. DOX, ^#^*p* < 0.05, ^##^*p* < 0.01 control Annexin V^+^ PI^+^ cells vs. CMCE or DOX, ^@^*p* < 0.05, ^@@^*p* < 0.01, ^@@@^*p* < 0.001 control Annexin V^-^ PI^+^ cells vs. CMCE or DOX treated cells.

Since the lower dose (1 μg/mL) of CMCE was ineffective in inducing cytotoxicity (**Figures 4A,B**), the other relevant doses of 10 and 30 μg/mL were chosen for carrying out mechanistic studies. In particular, cell cycle analysis revealed the presence of a sub-G1 peak in the DNA histogram distribution, indicating the accumulation of a late apoptotic population after CMCE treatment (10 and 30 μg/mL; 20 h). The sub-diploid population increased in a concentration-dependent manner (∼25 and 50%, respectively) in CMCE treated cells (**Figures 5A,B**). DOX (1 μg/mL) also induced sub-G1 apoptosis (∼26%) in HL-60 cells (**Figures 5A,B**). CMCE induced apoptosis of HL-60 cells was also confirmed by Annexin V-FITC/ PI staining. Annexin V is a Ca^2+^ dependent phospholipid-binding protein having a high affinity for apoptotic cells with exposed phosphatidylserine and is typically used in conjunction with PI to distinguish early from late apoptotic cells (Vermes et al., 1995). Healthy cells were negative for both Annexin V and PI. Apoptotic cells were Annexin V positive and PI negative. Late apoptotic and necrotic cells were positive both for Annexin V and PI (**Figures 5C,D**). CMCE treatment enhances the proportion of late apoptotic (Annexin V-FITC and PI-positive) and necrotic (PI-positive) cells at 10 μg/mL (∼8 and 12%, respectively) and 30 μg/mL (∼25 and 33%, respectively) concentrations (**Figures 5C,D**). DOX also significantly induced early apoptotic (∼18%), late apoptotic (∼15%) and necrotic (∼18%) cell populations (**Figures 5C,D**). HL-60 cells treated with CMCE showed increased numbers of Annexin V and PI-positive cells which indicative of late apoptosis. No significant sub-G1 peak and Annexin V-FITC and PI-positive cells were detected in vehicle-treated HL-60 cells.

### CMCE Induced Apoptosis Involves Loss of Mitochondrial Membrane Potential, Caspase-1, and IRAK

**FIGURE 6:**
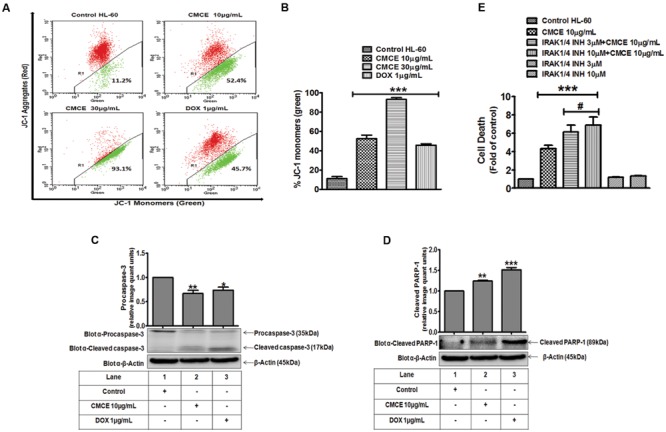
**Effect of CMCE on mitochondrial membrane potential, caspase-3 and PARP-1 activation and IRAK1/4 inhibition. (A)** Representative dot plot and **(B)** Bar diagram depicting % positive JC-1 monomers (green fluorescence) in HL-60 cells treated with CMCE (10 and 30 μg/mL) or DOX (1 μg/mL) for 20 h. Changes in transmembrane potential were assessed by flow cytometry using FL-1 (green) and FL-2 (red) channels. CMCE induced cleavage of **(C)** pro-caspase-3 and **(D)** PARP-1 in HL-60 cells. Cells treated with CMCE (10 μg/mL) or DOX (1 μg/mL) for 20 h and expression levels of pro-caspase-3 and PARP-1 were detected by Western blotting. β-Actin was used as an internal loading control. The blots are representative one of three similar experiments. **(E)** HL-60 cells were treated with 3 or 10 μM concentration of IRAK1/4 inhibitor with or without CMCE (10 μg/mL) for 20 h. Cell death was assessed by flow cytometry with Annexin V-PI staining. Values represent mean (*n* = 3) ± SEM; ^∗^*p* < 0.05, ^∗∗^*p* < 0.01, ^∗∗∗^*p* < 0.001 control vs. CMCE, CMCE+ IRAK1/4 inhibitor, or DOX treated cells; ^#^*p* < 0.05 CMCE vs. CMCE+IRAK1/4 inhibitor treated cells.

The HL-60 cells were treated with CMCE (10 and 30 μg/mL) or DOX (1 μg/mL) for 20 h and mitochondrial membrane potential loss was assessed by monitoring change in JC-1 fluorescence. CMCE induced apoptosis through the intrinsic pathway as was revealed by loss of mitochondrial membrane potential. A dose-dependent increase (∼52 and 93%, respectively) in JC-1 monomers (green fluorescence) was observed, indicating loss of mitochondrial membrane potential (**Figures 6A,B**). Similarly, DOX treatment also produced increases in JC-1monomers (∼46%) (**Figures 6A,B**). Since, caspases play a vital role in the initiation and execution of apoptosis and caspase-3 is an executioner caspase which cleaves its substrate PARP-1 (Nunez et al., 1998), expression of caspase-3 and PARP-1 was assessed in CMCE (10 μg/mL; 20 h) treated HL-60 cells. CMCE (10 μg/mL) or DOX (1 μg/mL) significantly induced procaspase-3 cleavage (∼0.7 fold each) and PARP-1 cleavage (∼1.2 and 1.5 fold, respectively) (**Figures 6C,D**). Therefore, disruption in the mitochondrial membrane potential further activated the procaspase-3 and PARP-1 cleavage via procaspase-9. The effect of the IRAK1/4 inhibitor on CMCE induced cytotoxicity was evaluated. Although the 3 and 10 μM concentrations of IRAK1/4 inhibitor alone had no significant effect on cell death similar concentrations of IRAK1/4 inhibitor (3 and 10 μM) potentiated (∼1.4 and 1.6 fold, respectively) CMCE (10 μg/mL) induced cell death (**Figure 6E**). This result implies that inhibition of IRAK1/4 sensitizes HL-60 cells to CMCE induced cytotoxicity.

## Discussion

The present study demonstrates, for the first time, the anti-inflammatory and cytotoxic activity of a novel non-carbonyl compound enriched *Curcuma longa* extract (CMCE) in various models of inflammation and cytotoxicity. The present modifications done to the *Curcuma longa* extract resulted in enrichment of non-carbonyl components and the compound I, while turmerones and curcumin were present in a negligible amount. The cytotoxic effect in the two AML cells lines and the anti-inflammatory effect in LPS-induced inflammation observed in THP-1 cells and *in vivo* demonstrates the therapeutic potential of this extract.

Although a controlled inflammatory response protects the host against invading pathogens, its dysregulation leads to several diseases such as cancer, atherosclerosis, rheumatoid arthritis and septic shock (Mogensen, 2009). By using THP-1 cells and a more physiologically relevant system of whole blood culture (Thurm and Halsey, 2005), the present study demonstrates that CMCE attenuates LPS-induced TNF-α and IL-1β production. Since the whole blood model closely imitates *in vivo* conditions, our findings also suggest the potential for effectiveness of CMCE *in vivo*.

LPS-induced TLR4 activation leads to the recruitment of MyD88, followed by subsequent induction of downstream signaling molecules like IRAK1, MAPKs, and NFκB (Medvedev et al., 2002). Moreover, phosphorylation of MAPKs (p38, JNK, and ERK) via various transcription factors regulates the production of inflammatory cytokines (Guha and Mackman, 2001). LPS mediated IκBα phosphorylation leads to its ubiquitination and degradation and facilitates translocation of NFκB to the nucleus (Kawai and Akira, 2007). The findings of the present study demonstrate that CMCE by reducing IRAK1, p38, JNK, ERK phosphorylation, and IκBα degradation may affect the activation of downstream transcription factors like AP-1, Elk-1, and NFκB, respectively and therefore attenuate inflammatory cytokines production in THP-1 cells. Thus, it is plausible that both the MAPK and the NF-κB pathway are key to the anti-inflammatory effect of CMCE. Inhibition of TLR4-mediated signaling can be achieved by either disruption of the TLR4-MyD88 complex or by downregulation of receptor itself (Methe et al., 2005; Piao et al., 2013). Expression and co-immunoprecipitation experiments revealed that both TLR4 expression and TLR4-MyD88 interaction were necessary for the observed anti-inflammatory effect of CMCE. Since, LPS-mediated activation of MAPKs and NFκB signaling encourages the binding of NFκB and AP-1 transcription factors to the promoter of the TLR4 gene and subsequently leads to its transcriptional up-regulation and cytokine production (Yan, 2006), our findings suggest that CMCE, by preventing the TLR4-MyD88 interaction and downregulating the TLR4 receptor attenuates LPS-induced pro-inflammatory cytokines production.

To determine its therapeutic relevance, CMCE was tested in an LPS-induced endotoxemia mouse model which closely mimics sepsis. Since, an earlier study from our laboratory showed that a hexane-soluble extract of *C. longa* had a lipid-lowering effect without altering liver enzymes (for 30, 100, and 300 mg/kg doses) (Singh et al., 2013), we used similar low, medium and high doses of CMCE to monitor activity against LPS-induced endotoxemia in a mouse model. In agreement with our *in vitro* results, plasma pro-inflammatory cytokines were significantly attenuated in the LPS-induced endotoxemia mouse model. Circulating cytokines released during septicemia promote blunted vascular responses due to impairment in endothelial function (Titheradge, 1999; Draisma et al., 2010). Endothelial dysfunction is caused by decreased eNOS and increased iNOS expression in the vessel wall (Titheradge, 1999; Draisma et al., 2010). Therefore, suppression of LPS-induced aortic iNOS expression and plasma nitrite production by CMCE could be partially responsible for the improved vascular relaxation when compared to groups treated with LPS alone.

TGA-induced peritonitis is characterized by distinct phases in which neutrophil influx occurs at an earlier time point (up to 24 h) followed by macrophage infiltration at later time points (48-72 h)(Melnicoff et al., 1989). In CMCE treated mice, the peritoneal lavage leukocytes were significantly reduced at 24 and 72 h suggesting that CMCE is likely to inhibit transmigration of both neutrophils and macrophages during the initiation and progression of the inflammatory response.

Given that molecular inhibition of the IRAK pathway by CMCE induced an anti-inflammatory effect and higher doses produced a cytotoxic effect in THP-1 cells, it was considered worthwhile to investigate the consequence of IRAK pathway inhibition by CMCE on cytotoxicity in cancer cell lines. Previous studies indicate an association of myeloid leukemia with hyperactivation of the IRAK pathway (Rhyasen and Starczynowski, 2015). At the same time, a small molecule possessing anti-inflammatory and anti-tumor effects was found to inhibit IRAK1 (Joh et al., 2011; Rhyasen and Starczynowski, 2015). Several FDA-approved drugs demonstrated enhanced cytotoxicity when combined with an IRAK1/4 inhibitor (Li et al., 2015). IRAK1 is also reported to drive breast cancer cell metastasis, and its inhibition overcomes paclitaxel-induced resistance (Wee et al., 2015). Therefore, HL-60, MCF-7, and MDA-MB-231 cell lines were used to test the effect of CMCE on cytotoxicity. Also in previous studies, *C. longa* extracts containing curcumin and turmerones had been tested for similar activities in these cell lines (Aratanechemuge et al., 2002; Sandur et al., 2007; Yue et al., 2010). These cell lines are also a part of the NCI-60, the cell line panel recommended for anti-cancer screening (Ikediobi et al., 2006).

Loss of mitochondrial potential and activation of the intrinsic pathway by CMCE is important since activating the intrinsic, or the mitochondrial pathway of apoptosis is one of the therapeutic approaches in AML (Kadia et al., 2016). ABT-199 showed significant mitochondrial-dependent activity in leukemia cell lines, primary murine xenografts, and primary samples of patients with AML including AML stem and progenitor cells (Pan et al., 2014; Kadia et al., 2016). Current and future AML therapy may involve the development of agents that target specific mutant driver enzymes or proteins and drugs like CPX-351, SGN-CD33A, and ABT-199 whose mechanism of action and efficacy may be independent of mutational complexity (Stein and Tallman, 2016). One effective strategy could be to combine drugs with the non-overlapping mechanism of actions with targeted molecular therapy agents (Stein and Tallman, 2016). The combination studies for ABT-199 are already underway (Kadia et al., 2016). Therefore, it will be interesting to assess the effect of CMCE on AML patient samples in combination with known and molecular targeted agents.

Although inhibition of the IRAK pathway by CMCE induced an anti-inflammatory effect, IRAK1/4 inhibition alone was not sufficient to induce cell death in HL-60 cells. However, IRAK inhibition potentiated the cytotoxic activity of CMCE in HL-60 cells. This means that mere inhibition of the IRAK pathway is not sufficient to induce cell death. Therefore, it can be speculated that enhanced sensitivity to CMCE induced cell death in the presence of IRAK1/4 inhibitor can be due to enhanced cooperativity between the IRAK dependent and independent signaling pathways activated by CMCE. At the same time, effective inhibition of IRAK by the IRAK1/4 inhibitor may also account for an efficient cytotoxic effect of CMCE at the tested dose. Therefore, CMCE due to its potential to inhibit the IRAK pathway may have the dual potential of sensitizing the cells to cytotoxicity and also participate in the phenomenon directly. Although inflammation is associated with the development of several cancers like colitis-associated colorectal cancer (Kim and Chang, 2014), it will be interesting and worthwhile to investigate the effect of CMCE in a model of cancer where inflammation is a major underlying mechanism.

## Conclusion

*Curcuma longa* L. extract (CMCE) was found to exert anti-inflammatory effects *in vitro* and *in vivo*. It also promoted a cytotoxic effect in cancer cells. CMCE modulated the TLR4-MyD88-IRAK-MAPK-NFκB pathway in THP-1 cells and regulated inflammatory cytokine production. CMCE ameliorated LPS-induced adverse effects in mice. In HL-60 cells, the cytotoxic effect was mediated by the intrinsic pathway of apoptosis, leading to caspase-3 activation, which was further enhanced by IRAK inhibition. Thus, CMCE sensitized the cells to cytotoxicity and also participated in the phenomenon directly. Therefore, molecular inhibition of the IRAK pathway by CMCE regulated inflammation and cytotoxicity. For these reasons, CMCE may have beneficial therapeutic applications in inflammatory and proliferative disorders.

## Author Contributions

MR was responsible for the planning, execution of all experiments and preparation of the manuscript. PM, SS, and VS were responsible for endothelial dysfunction and TGA-induced mice peritonitis model experiments. HA and AD were responsible for the preparation, isolation, characterization, and bioavailability study of CMCE. MD provided critical inputs for the experiments. MB was responsible for the conceptualization, planning, execution and troubleshooting of the experiments, preparation of the manuscript and the financial support.

## Conflict of Interest Statement

The authors declare that the research was conducted in the absence of any commercial or financial relationships that could be construed as a potential conflict of interest.

## Abbreviations

AML: Acute myeloid leukemia
DOX: Doxorubicin
IRAK1: Interleukin-1 receptor-associated kinase 1
LPS: Lipopolysaccharide
MAPKs: Mitogen-activated protein kinases
SRB: Sulforhodamine B
TGA: Thioglycollate
TLRs: Toll-like receptors
