# Supplementary material for: A Standardized Chemically Modified Curcuma longa Extract Modulates IRAK-MAPK Signaling in Inflammation and Potentiates Cytotoxicity
Source: Front Pharmacol. 2016 Jul 25;7:223. doi: 10.3389/fphar.2016.00223 (PMC4959270; doi:10.3389/fphar.2016.00223)
Supplement: Supplementary file 1 [file Image_1.PDF]

Supplementary Material

IRAK Mediates Anti-inflammatory and Cytotoxic Potential of a Standardized Chemically Modified *Curcuma longa* Extract

Minakshi Rana[1], Preeti Maurya[1], Sukka Santosh Reddy[1], Vishal Singh[1], Hafsa Ahmad[2], Anil Kumar Dwivedi[2], Madhu Dikshit[1] and Manoj Kumar Barthwal[1]*

*** Correspondence:** Dr. Manoj Kumar Barthwal: manojbarthwal@cdri.res.in

Preparation of standard and quality control samples

A stock solution of CMCE was prepared in methanol to give a final concentration of 200 μg/mL. A calibration range of standard solutions (2-150 μg/mL) was obtained by serial dilution method with methanol. All the solutions were stored at -20°C and were brought to room temperature before use. To prepare the standard plasma calibration samples, 50 μL of standard solutions were added to 200 μL of blank plasma. The mixture was then treated following sample extraction procedure described below. The final standard plasma concentrations were 0.5-50 μg/mL for CMCE (Jain et al., 2007).

Supplementary Figures and Figure Legends

Figure S1

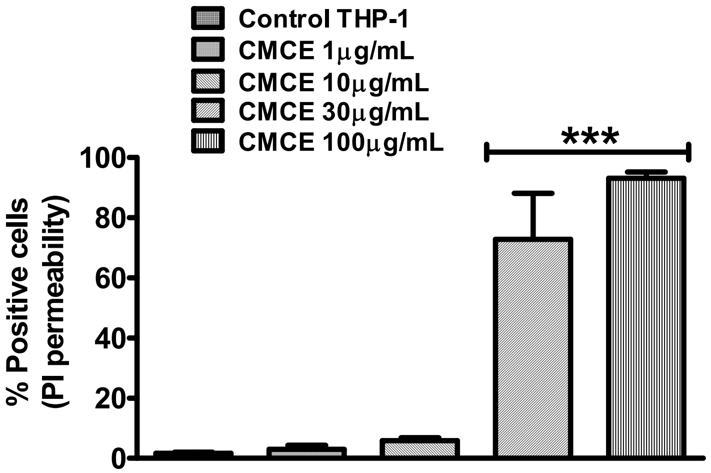

Supplementary Figure S1: Effect of CMCE on cell viability in THP-1 cells

THP-1 cells treated with different concentrations of CMCE for 14 h and cell viability was examined by propidium iodide staining by flow cytometry. Values represent as the mean (at least n=3) ± SEM; ***p< 0.001 control vs. CMCE treated cells.

Figure S2

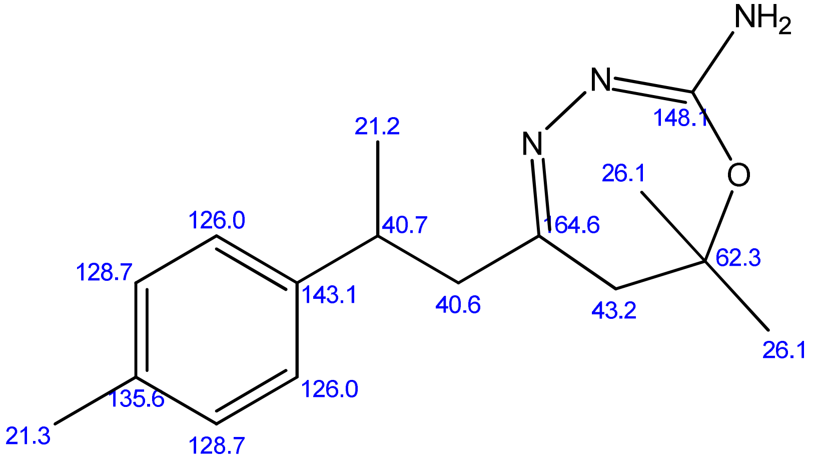

Supplementary Figure S2: Chemical structure of marker compound 7,7-dimethyl-5-(2-p-tolylpropyl)-6,7-dihydro-1,3,4-oxadiazepin-2-amine; Compound (I)

^{1}H NMR (400 MHz, DMSO-d$_6$): δ 7.13(d, 2H, J= 7.68), δ 7.08(d, 2H, J= 7.56), δ 5.93 (S, 2H), δ 3.01 (dd, 1H, J=6.90), δ 2.5 (m, 4H), δ 2.24(s, 3H) , δ 1.31 (s, 3H), δ 1.23(s, 3H), δ 1.18 (d, 3H, J=6.72)

^{13}C NMR (400 MHz, DMSO-d$_6$): 155.41, 153.28, 142.96, 135.04, 128.86, 126.69, 61.66, 50.85, 37.98, 36.55, 26.00, 25.78, 22.29, 20.59

IR (neat) υ (cm^{-1}) 3411.7, 3015.2, 1667.2, 1564.5, 1514.7, 1448.0, 1216.1, 1054.7, 759.7, 669.1, 544.7

Mass m/z 274.5(M^{+}+1), (M^{+}+2) 275.6, HRMS m/z 274.1932 (M^{+}+1), 275.1956 (M^{+}+2)

Figure S3

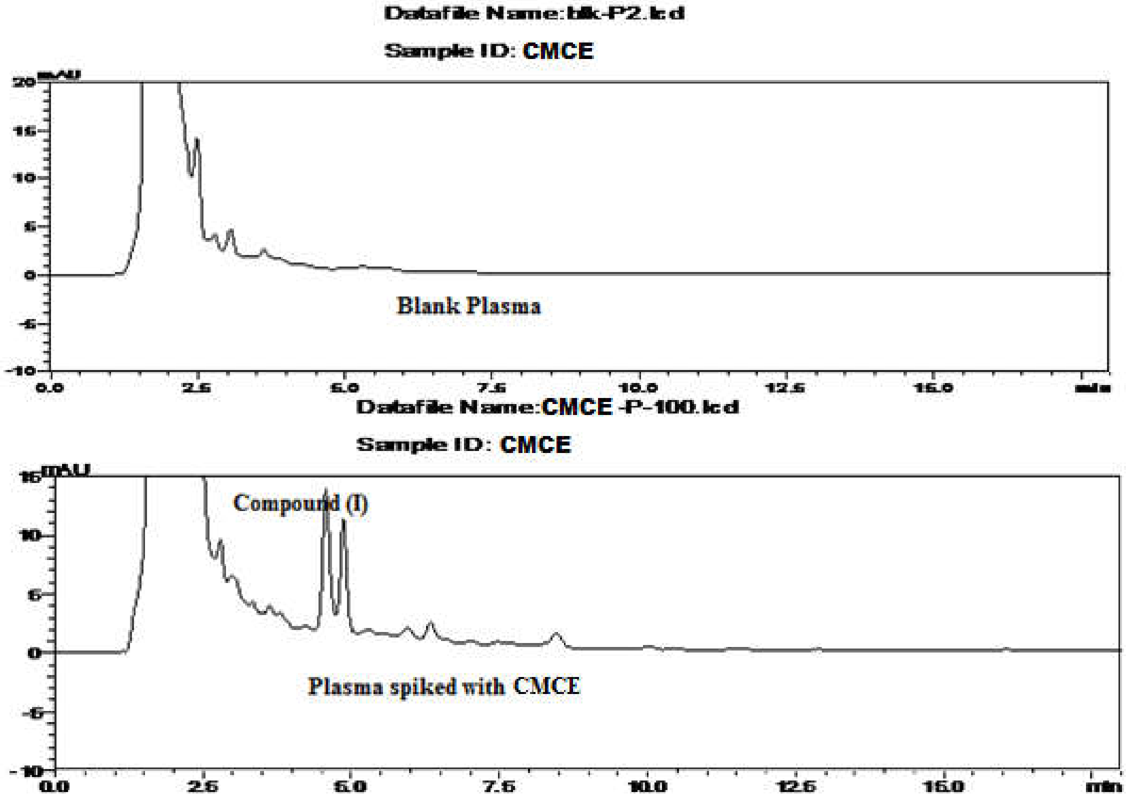

Supplementary Figure S3: The HPLC chromatogram of blank plasma and plasma spiked with CMCE on RP 18e Durashell (250 x 4.6mm, 3µm; 100 Å) column using mobile phase of acetonitrile: water (70:30).

Figure S4

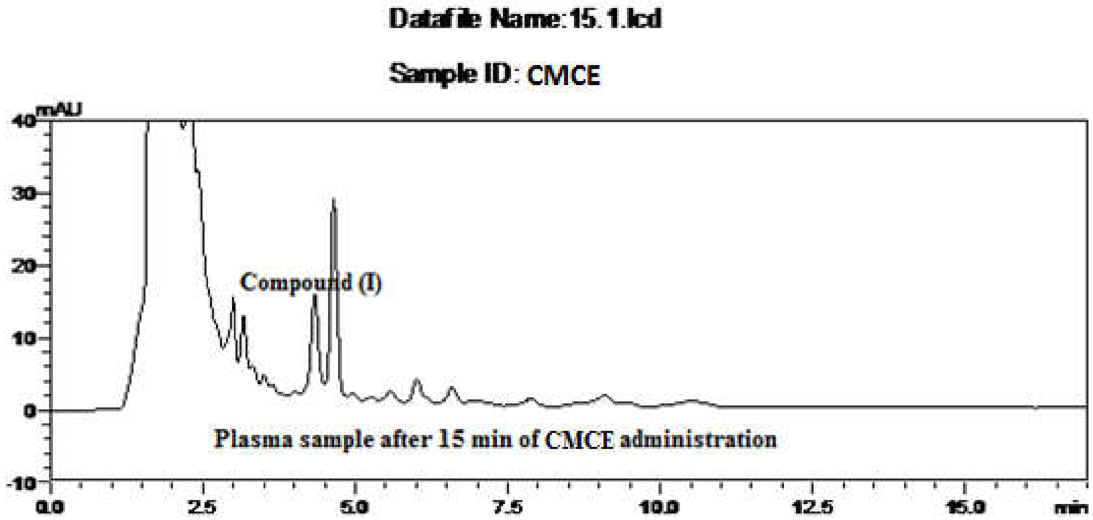

Supplementary Figure S4: The HPLC chromatogram of plasma after 15 min of CMCE administration on RP 18e Durashell (250 x 4.6mm, 3µm; 100 Å) column using mobile phase of acetonitrile: water (70:30).

References

Jain, V., Prasad, V., Pal, R., and Singh, S. (2007). Standardization and stability studies of neuroprotective lipid soluble fraction obtained from Curcuma longa. *J Pharm Biomed Anal* 44, 1079-1086.
